# Microstructure and Friction Properties of CoCrFeMnNiTi_x_ High-Entropy Alloy Coating by Laser Cladding

**DOI:** 10.3390/ma15134669

**Published:** 2022-07-03

**Authors:** Pengfei Liu, Wudong Si, Dabin Zhang, Sichao Dai, Benchi Jiang, Da Shu, Lulu Wu, Chao Zhang, Meisong Zhang

**Affiliations:** 1 School of Mechanical Engineering, Guizhou University, Guiyang 550025, China; liupengfei0531@163.com; 2 School of Mechanical Engineering, Anhui Polytechnic University, Wuhu 241000, China; s303537841@gmail.com (W.S.); dscwudi666@163.com (S.D.); benchi@ahpu.edu.cn (B.J.); wull@ahpu.edu.cn (L.W.); 3 Nano and Molecular Systems Research Unit, Faculty of Science, University of Oulu, FIN-90014 Oulu, Finland; 4 School of Materials Science and Engineering, Nanjing Institute of Technology, Nanjing 211167, China; zhangchao@njit.edu.cn; 5 Anhui Honggu Laser Co., Ltd., Wuhu 241299, China; meisong202206@163.com

**Keywords:** laser cladding, high-entropy alloy, CoCrFeMnNiTi, microstructure, friction and wear

## Abstract

To enhance the friction and wear properties of 40Cr steel’s surface, CoCrFeMnNi high-entropy alloy (HEA) coatings with various Ti contents were prepared using laser cladding. X-ray diffraction (XRD), scanning electron microscopy (SEM), and energy dispersive spectroscopy (EDS) were used to characterize the phase composition, microstructure, and chemical composition of the samples. The findings demonstrated that the CoCrFeMnNiTi_x_ HEA coatings formed a single FCC phase. Fe_2_Ti, Ni_3_Ti, and Co_2_Ti intermetallic compounds were discovered in the coatings when the molar ratio of Ti content was greater than 0.5. The EDS findings indicated that Cr and Co/Ni/Ti were primarily enriched in the dendrite and interdendrite, respectively. Ti addition can effectively enhance the coating’s mechanical properties. The hardness test findings showed that when the molar ratio of Ti was 0.75, the coating’s microhardness was 511 HV0.5, which was 1.9 times the hardness of the 40Cr (256 HV0.5) substrate and 1.46 times the hardness of the CrCrFeMnNi HEA coating (348 HV0.5). The friction and wear findings demonstrated that the addition of Ti can substantially reduce the coating’s friction coefficient and wear rate. The coating’s wear resistance was the best when the molar ratio of Ti was 0.75, the friction coefficient was 0.296, and the wear amount was 0.001 g. SEM and 3D morphology test results demonstrated that the coating’s wear mechanism changed from adhesive wear and abrasive wear to fatigue wear and abrasive wear with the increase in Ti content.

## 1. Introduction

40Cr steel has excellent strength, stiffness, and plasticity and is extensively employed in mechanical parts, such as midload gears, crankshafts, and oil pump rotors. Wear is one of the primary failure modes of 40Cr steel in service. Wear consumption results in a large amount of energy and material consumption. Enhancing the wear resistance of parts has attracted considerable attention. Generally, heat treatment can be used to enhance 40Cr steel’s wear resistance, but owing to the process’s complexity and difficulty in controlling time and temperature, it will waste energy [1,2,3,4,5]. Laser cladding, an advanced surface modification technology, is crucial for rapidly heating and curing metal powder into coatings using a high-energy laser to repair and enhance the surface of parts [6,7]. Laser cladding has special advantages such as a low dilution rate, low thermal impact, a fine cladding layer microstructure, and a good metallurgical combination compared with other technologies [5]. Zhang et al. [8] prepared Ti_3_AlC_2_ reinforced Co-based alloy coatings to repair H13 steel, and verified that the coating had outstanding tribological properties. Wang et al. [9] fabricated the Stellite-6-WC-12Co composite coating on AISI H13 tool steel by Laser cladding, which had excellent wear resistance at high temperatures.

A high-entropy alloy (HEA) is an emerging alloy system. It is characterized by high strength [10], high hardness [11], corrosion resistance [12], high wear resistance [13], and outstanding resistance to high-temperature softening [14] due to its unique high-entropy effect, lattice distortion effect, sluggish diffusion effect, and cocktail effect [15]. The wear resistances of AlCrFeNiW_0.2_Ti_0.5_ coatings were investigated by Liang et al. [16]. It was found that the AlCrFeNiW_0.2_Ti_0.5_ coating showed more excellent tribological performances than Q235 steel and SUS304, which was mainly due to its higher hardness and the formation of Mg(OH)_2_, CaCO_3_, metal oxides, and hydroxides, and the formation of a protective tribo-film on the worn surface. Zhang et al. [17] have studied the wear resistance of a FeNiCoCrTi_0.5_ HEA coating, finding that the wear resistance of the coating was much higher compared to 45 steel. Among most HEAs, the tensile strength and fracture toughness of the CoCrFeMnNi HEA at low temperatures reach 1280 MPa and 300 MPa·m^1/2^, respectively, which has attracted the interest of numerous scholars [18,19]. To enhance mechanical properties, increasing the CoCrFeMnNi’s strength and hardness by adding one or more elements into the system and regulating the content of the elements is an effective modification approach. Hsu et al. [20] prepared a CoCrFeMnNiAl_x_ coating using the magnetron sputtering approach. The phase changed from FCC, FCC + BCC, and BCC successively with an increase in Al content, and hardness increased from 5.71 to 8.74 GPa. Kumar et al. [21] prepared CoCrFeMnNiAl_x_ using vacuum arc melting technology. The phase changed from a single FCC to an FCC + B2 phase with an increase in Al content, and hardness increased from 1.3 to 2.2 GPa. Huang et al. [22] prepared (CoCrFeMnNi)100_x_Mo_x_ HEA coatings. The phase changed from FCC to an amorphous structure with an increase in Mo content to 14.6 at.%, and the coating’s yield strength increased from 1.69 to 5.16 GPa. Yeong et al. [23] prepared a CoCrFeMnNiC_x_ HEA. The strength increased from the original 253 to 1062 MPa with an increase in C content to x = 0.7. Fang et al. [24] prepared CoCrFeMnNiV_x_ coatings, the V content increased to 1.1, and the film’s hardness ranged from 6.8 to 8.7 GPa.

A Ti element is a silver-white metal with high strength, easy processing, and low- and high-temperature resistance. Like an Al atom, it has a large atomic radius that can aggravate lattice distortion and can enhance solid solution strengthening. Meanwhile, Ti can dissolve in an FCC solid solution, resulting in a phase and microstructure transformation [25]. Wang et al. [26] investigated the Ti element content’s effect on a CoCrFeNiTi_x_ HEA and discovered that a Ti element could not only strengthen FCC’s lattice distortion in the matrix phase but also enhance the growth of secondary dendrite arms and reduce the spacing of secondary dendrite arms, thus increasing the cladding layer’s hardness from 310 to 830 HV. Zhang et al. [27] prepared a CoCrFeMnNiTi_x_ coating using plasma cladding and discovered that with an increase in Ti element content, the microstructure of the coatings from bottom to top changed from a single columnar crystal group to an equiaxed crystal and “snowflake” structure. Hsu et al. [28] prepared a CoCrFeMnNiTi high-entropy alloy coating using a co-sputtering approach with Ti as the target material; they discovered that Ti could transform CoCrFeMnNi from a single FCC solid solution structure to an amorphous phase structure, and the coating’s hardness increased from 6.62 to 8.99 GPa with an increase in Ti element content. Wang et al. [29] observed BCC and intermetallic compounds in (CoCrFeMnNi)_85_Ti_15_, and hardness was substantially enhanced to approximately 910 HV. In conclusion, the introduction of Ti can effectively enhance an HEA’s mechanical properties. Although numerous scholars have researched a Ti element-modified HEA, the influence of Ti on the friction and wear properties of the HEA and the mechanism of friction and wear were still insufficient, and it is crucial to perform further research.

In this study, the influences of Ti addition on the microstructural aspects of the CoCrFeMnNi HEA prepared with laser cladding approaches were examined. Furthermore, the wear behavior and wear mechanism of the CoCrFeMnNi HEA were investigated.

## 2. Materials and Methods

### 2.1. Starting Powders

**Figure 1:**
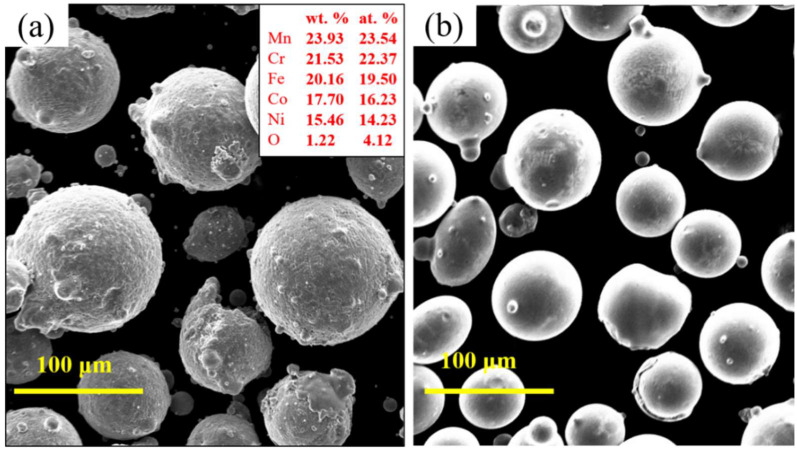
SEM morphology of the starting powders: (**a**) CoCrFeMnNi and (**b**) Ti.

The HEA powders of CoCrFeMnNi (purity of >99.8 and 90 ≤ particle size ≤ 105 μm; Luo Hong Technology Co., LTD, Shijiazhuang, China) and Ti powder (purity of >99.8 and 25 ≤ particle size ≤ 50 μm; Luohong Technology) were employed as starting materials. Figure 1 illustrates the two powders’ morphology. The overall morphology of the two powders showed high sphericity. Thus, the powders have excellent fluidity in the cladding process and effectively avoid powder agglomeration. In this experiment, the CoCrFeMnNiTi_x_ (x: molar ratio; x = 0, 0.25, 0.5, 0.75, and 1.0) HEA coatings were fabricated using laser cladding by adding various molar ratios of Ti to the CoCrFeMnNi high-entropy alloy. Table 1 shows the powder element ratios. The prepared powder was mixed using a vertical planetary ball mill (YXQM-2L, Miqi Instrument Equipment, Changsha, China) and dried in a vacuum for 2 h to ensure fluidity. The planetary ball milling process parameters were as follows: a rotation speed of 300 r/min and a ball milling time of 2 h. Ceramic balls were employed as grinding balls with a ball-to-powder ratio of 3:1.

### 2.2. Sample Preparation

**Figure 2:**
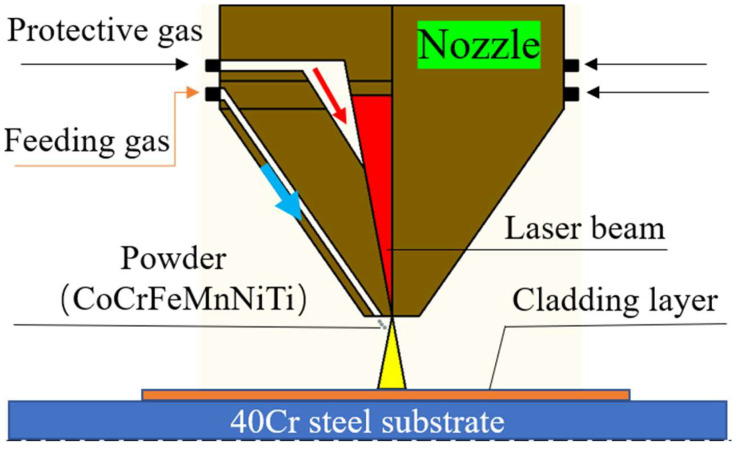
Diagram of the working principle of the laser head.

In this experiment, the CoCrFeMnNiTi_x_ (x: molar ratio; x = 0, 0.25, 0.5, 0.75, and 1.0) HEA coatings were fabricated using laser cladding. The 40Cr steel with a size of 100 mm × 100 mm × 10 mm was chosen as the substrate, which was polished and cleaned with alcohol to remove oil stains before the experiment. CoCrFeMnNiTi_x_ HEA coatings were prepared on the 40 Cr steel using laser cladding with a coaxial powder feeding approach. A six-axis robot (RS050N, Kawasaki, Osaka, Japan) equipped with a fiber laser (IPG8000, Photonics, Long Island, NY, USA) with a maximum output of 8000 W was employed for laser cladding experiments. Furthermore, the working chamber was filled with high-purity argon during the laser cladding process to prevent the molten pool metal from oxidation. The working principle of the laser head is shown in Figure 2. The processing parameters for laser cladding were as follows: a laser power of 1500 W, a scanning speed of 8 mm/s, a powder feeding speed of 1.2 r/min, a defocus distance of 40 mm, and an overlap rate of 50%. Argon carrier gas at a flow rate of 10 L/min was employed for the coaxial powder delivery and shielding gas. For simplicity, CoCrFeMnNiTi_x_ (x = 0, 0.25, 0.5, 0.75, and 1.0) HEA coatings are represented as Ti_0_, Ti_0.25_, Ti_0.5_, Ti_0.75_, and Ti_1_, respectively.

### 2.3. Material Properties

#### 2.3.1. Characterization Methods

The coating’s phase composition was identified using an X-ray diffractometer (XRD; D8 advanced, Bruker, Germany) with a voltage of 40 kV, current of 30 mA, scanning speed of 5°/min, and scanning range from 20° to 100°. The coatings’ microstructure and chemical composition were observed and analyzed using a scanning electron microscope (SEM; EM30AXP, COXEM, Daejeon, Korea) equipped with an energy dispersive spectrometer (EDS) with an acceleration voltage of 20 kV and a working distance of 11.0.

#### 2.3.2. Micro—Vickers Hardness 

The coatings’ microh ardness was measured using a microhardness tester (HV-1000, Xinghui Electronics Xinhui Co., Ltd., Jiangmen, China) with a load of 500 g and a duration time of 10 s. According to Formula (1) [30], the indentation’s diagonal length was measured. The indentation was made every 0.1 mm, and each indentation was tested three times, taking the arithmetic average as the final hardness value.

(1) $$HV=0.1891\times \frac{F}{d^{2}}$$

where *F* represents the pressure of the test load (N), and *d* represents the arithmetic mean of the diagonals of the indentation cross (mm).

#### 2.3.3. Nanoindentation

The coatings’ nanoindentation properties were measured using the nanoindentation (N.I.) test (G200, KLA-Tencor, Milpitas, CA, USA). The maximum load was 50 mN, and the dwelling time at the maximum load was 10 s. The coating’s nanohardness and elastic modulus were recorded to examine the friction and wear mechanisms.

#### 2.3.4. Wear Tests

In the case of dry sliding, a pin-disc friction and wear testing machine (HT-600, Zhongke KaiHua Co., Ltd., Lanzhou, China) was employed to conduct wear experiments on the coating. The speed was set at 500 r/min, the test force was 40 N, the test time was 1200 s, and the test torque was 1.366 N·m. Wear samples were prepared in the form of disks with a diameter of approximately 25 mm and a thickness of approximately 10 mm. Si_3_N_4_ pellets with a hardness of 75 HRC and a diameter of 50 HRC were employed as the dual samples. The wear surface morphology and wear mechanism were examined using SEM and a three-dimensional noncontact morphometer (PS50, NANOVEA, Irvine, CA, USA).

## 3. Result and Discussion

### 3.1. Macroscopic Morphology and Phase Composition

**Figure 3:**
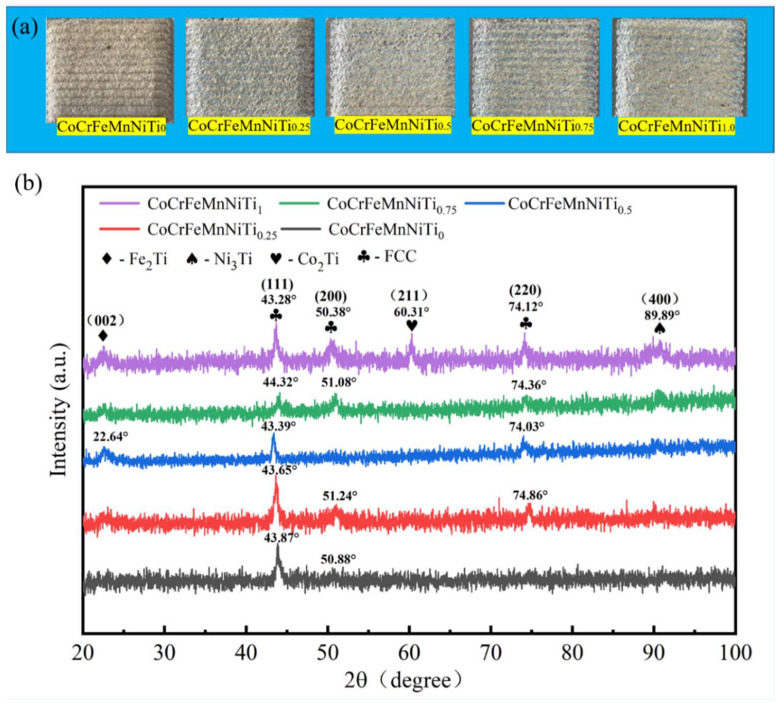
(**a**) Surface topography of CoCrFeMnNiTi_x_ coatings and (**b**) XRD pattern of CoCrFeMnNiTi_x_ coatings.

As shown in Figure 3a, a CoCrFeMnNiTi_x_ HEA coating with various Ti element contents was prepared using the process parameters optimized in our previous stage. It can be seen that the CoCrFeMnNiTi_x_ coatings were well-formed without visible pores and cracks, demonstrating that the laser parameters were properly selected. Figure 3b illustrates the XRD patterns of the CoCrFeMnNiTi_x_ HEA. The CoCrFeMnNi HEA coating was composed of a single FCC phase because the elements in the coatings were mutually dissolved at high temperatures and formed a single solid solution under the action of the high-entropy effect. With the addition of Ti, the characteristic diffraction peaks were 43.87°, 43.65°, 43.39°, 43.32°, and 43.28°, and the lattice constants were 3.583, 3.597, 3.601, 3.606, and 3.612 Å, respectively [23]. This demonstrated that the FCC phase lattice constant increased in the coating with an increase in Ti content because Ti (1.47 Å) has a higher atomic radius than the atomic radius of Co (1.25 Å), Cr (1.25 Å), Fe (1.26 Å), Mn (1.27 Å), and Ni (1.24 Å). When the Ti atom was solubilized in the alloy, it migrated to the alloy system, resulting in lattice distortion and lattice expansion. Fe_2_Ti (PDF #15-0336), Ni_3_Ti (PDF #51-1169), and Co_2_Ti (PDF #05.0719) intermetallic compounds precipitated when the atomic ratio of the Ti element exceeded 9.09%, because the solubility of Ti in the CoCrFeMnNi solid solution alloy is limited, and further addition of Ti will result in the main phase FCC’s instability.

### 3.2. Microstructure

**Figure 4:**
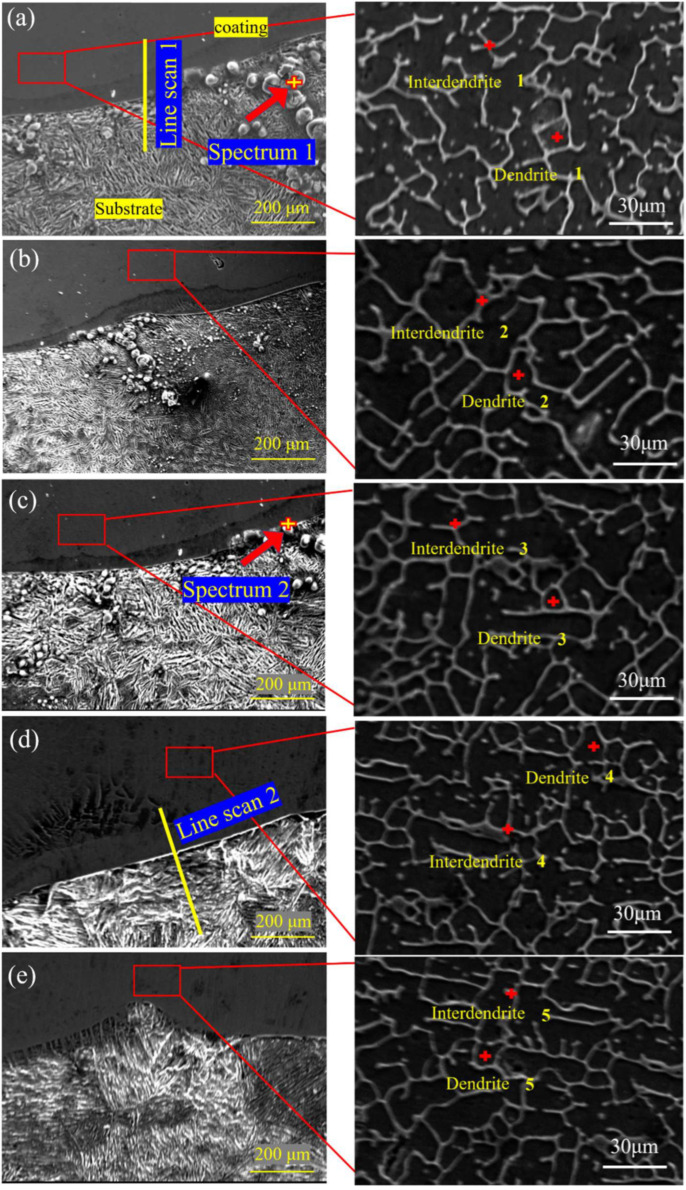
SEM diagram of the microstructure of the CoCrFeMnNiTi_x_ high—entropy alloy cladding section: (**a**) Ti_0_, (**b**) Ti_0.25_, (**c**) Ti_0.5_, (**d**) Ti_0.75_, and (**e**) Ti_1_.

**Figure 5:**
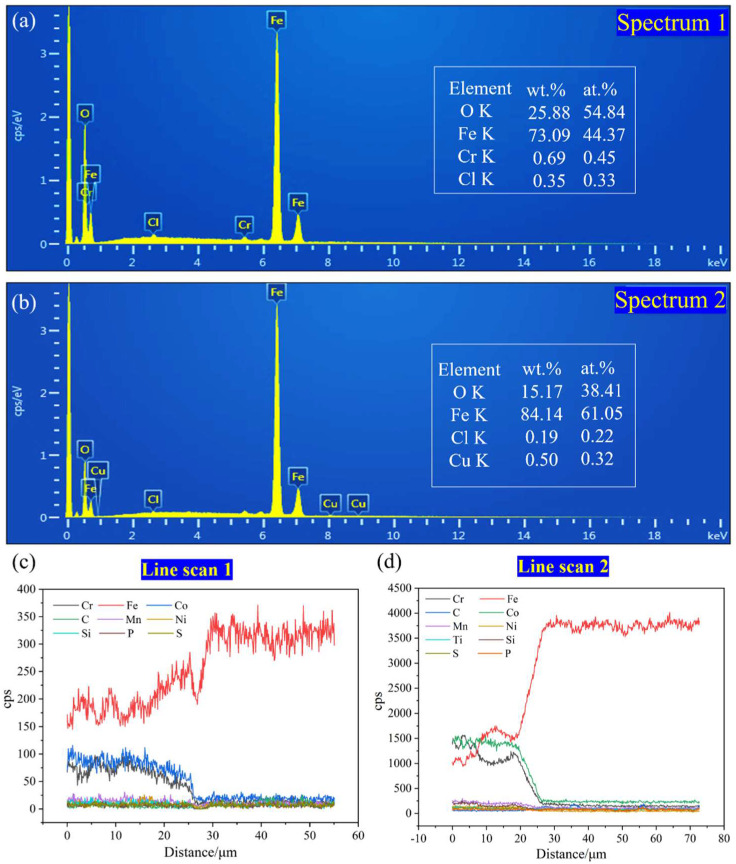
(**a**) Dot scanning spectrum of the Ti_0_ coating, (**b**) dot scan spectrum of the Ti_0.5_ coating, (**c**) line sweep spectrum of the Ti_0_ coating, (**d**) line sweep spectrum of the Ti_0.75_ coating.

Figure 4 shows the SEM diagram of a section of the CoCrFeMnNiTi_x_ coatings. There was an interface with a bright white band between the coating and substrate, which was tightly bonded without visible defects, showing that there was good metallurgical bonding between the coating and substrate. A certain number of particles appeared near the substrate’s interface, and point scanning was performed to obtain the spectra, as shown in Figure 5a,b. The particles were primarily composed of O and Fe elements, which was due to FeO being generated at the interface during the laser cladding process. The particles at the interface gradually disappeared with the increase in Ti content, demonstrating that the introduction of Ti can inhibit the formation of oxides. Furthermore, the cross-sections of the Ti_0_ and Ti_0.75_ coatings were scanned. Figure 5c,d show the line scanning spectra. Some elements of the substrate entered the coating. Because the content of the elements diluted from the substrate to the coating is not high, there was no serious infiltration phenomenon, showing that the technological parameters of the cladding were appropriate. From the coatings’ local magnification shown in Figure 4a–e, the coating presented a typical dendrite structure. The increase in Ti content resulted in the further growth of the secondary dendrite arms and a decrease in secondary dendrite arm spacing. As shown in Table 2, EDS measured the chemical composition of the different areas of the alloy. Co, Ni, and Ti were primarily enriched in the interdendritic region, and Cr was primarily enriched in the dendrite region. The deposition of the intermetallic compounds, consisting of Fe_2_Ti, Ni_3_Ti, and Co_2_Ti, occurred in the region enriched with Co, Ni, and Ti with the addition of Ti according to the XRD shown in Figure 3b. However, the melting point of Cr (1907 °C) is higher than that of Mn (1245 °C), Ni (1455 °C), Co (1495 °C), and Fe (1538 °C), and elements with a higher melting point are preferentially solidified into dendrites during solidification. The binary mixing enthalpy values of Ti with Co and Ni were −28 and −35 kJ/mol, respectively, which were higher than the binary mixing enthalpy values of Ti with Fe, Mn, and Cr of −17, −8, and −7 kJ/mol. Thus, Ti is easy to solution to dissolve in the base phase of CoCrFeMnNi. Intermetallic compounds such as Ni_3_Ti and Co_2_Ti are formed by increasing lattice distortion and reacting with Co and Ni to change the coating’s mechanical properties.

### 3.3. Microhardness

**Figure 6:**
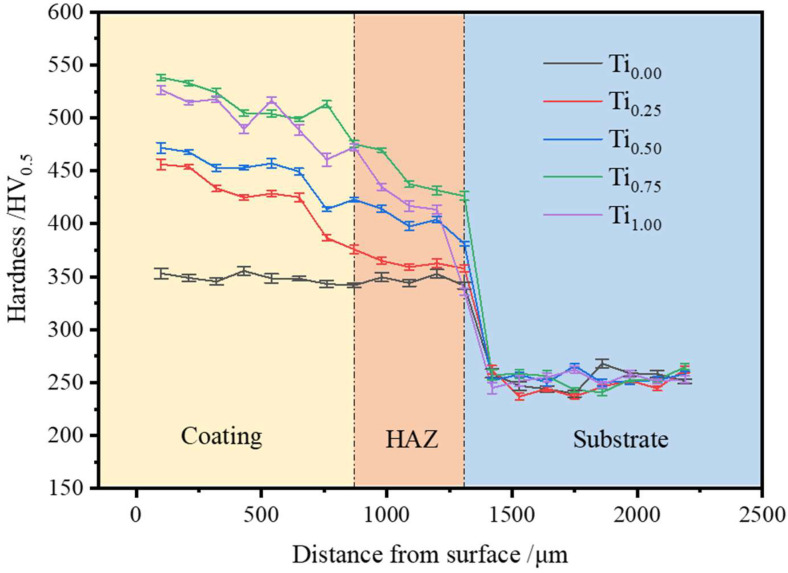
Microhardness distribution curves of CoCrFeMnNiTi_x_ alloy coatings with different Ti contents.

Figure 6 illustrates CoCrFeMnNiTi_x_ HEA coatings’ hardness distribution with various Ti contents from the top to bottom of the coating. With the increase in Ti content, the HEA’s microhardness increased first and then decreased. The five coatings’ average microhardness values were 348 ± 6.12, 423 ± 5.23, 448 ± 7.95, 511 ± 4.86, and 498 ± 6.19 HV0.5, respectively. The average microhardness of the Ti_0.75_ coating was 1.46 times that of the Ti_0_ coating and 1.9 times that of the substrate (~256 HV0.5); with the increase in Ti content, the lattice distortion effect and solid solution strengthening became more visible, thus maintaining relatively high hardness. Simultaneously, the larger size of Ti not only intensified the lattice distortion effect but also enhanced the formation of intermetallic compounds in the microstructure and inhibited grains’ growth and crystallization. With a further increase in Ti content, when the molar ratio increased to x = 1, the Ti atom’s solubility decreased, and the hardness of the coating decreased slightly.

### 3.4. Wear Properties

**Figure 7:**
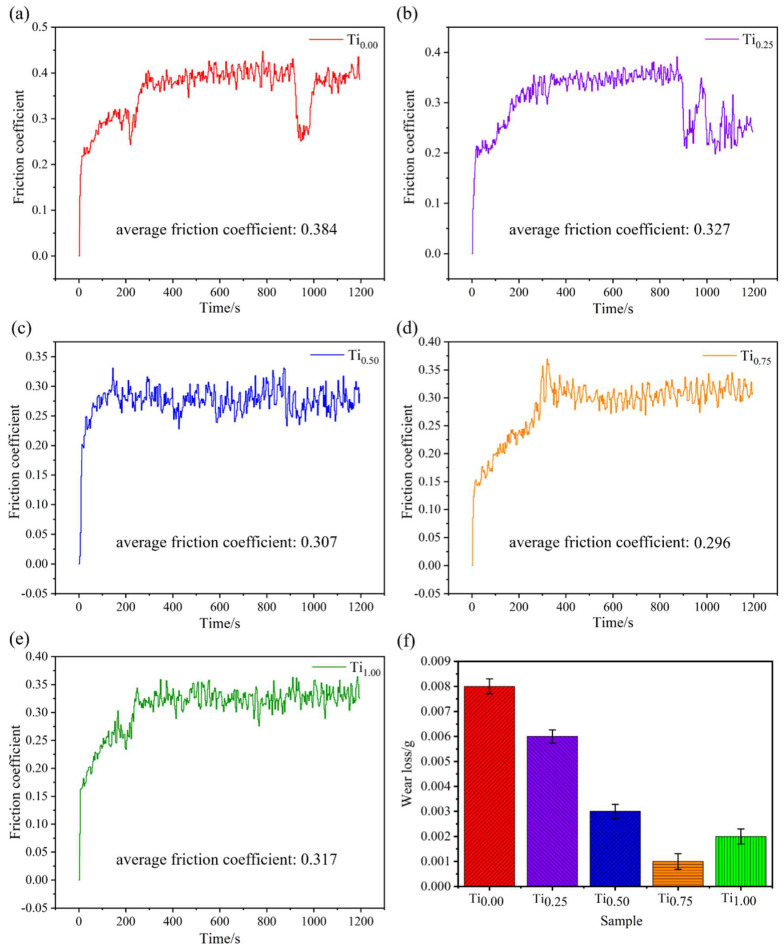
Friction coefficient curves of CoCrFeMnNiTi_x_ alloy coatings with different Ti contents: (**a**) Ti_0_, (**b**) Ti_0.25_, (**c**) Ti_0.5_, (**d**) Ti_0.75_, (**e**) Ti_1_, and (**f**) wear loss of CoCrFeMnNiTi_x_ high-entropy alloy coatings with different Ti contents.

Figure 7 illustrates the friction coefficient curve and wear number of the CoCrFeMnNiTi_x_ HEA coatings. The friction coefficient of the CoCrFeMnNiTi_x_ HEA coatings with various Ti contents increased sharply to the range of 0.25–0.4 at the initial running-in stage because the microconvexity on the surface of the friction pair contacted each other at the initial running-in stage and there was high contact stress, which caused severe wear and tear, so the coefficient of friction considerably increased. The contact area of the surface friction pair ball increased when the friction was in the middle-later stage, and the friction coefficient did not increase and was in a stable fluctuation stage. As shown in Figure 7a,b, Ti_0_ and Ti_0.25_ coatings have a large drop in the time range of 900~1000 s. Due to the poor wear resistance of the surface, plastic deformation occurs on both sides of the cladding layer surface in the friction process, so that the wear debris accumulates on both sides, and the contact ball and wear mark contact is shallow. The wear quantities were 0.008, 0.006, 0.003, 0.001, and 0.002 g, respectively. The wear loss reached the lowest value of 0.001 g when the molar ratio increased to x = 0.75, which was reduced by 0.007 g compared with the Ti_0_ coating, and the friction coefficient of Ti_0.75_ coating reached the lowest value of 0.296, primarily because the increase in hardness can enhance the coating’s wear resistance. Furthermore, the strengthening effect of the Fe_2_Ti and Ni_3_Ti intermetallic compounds in the coating enhanced the coating surface’s wear resistance, which showed that the introduction of Ti can enhance the wear resistance.

**Figure 8:**
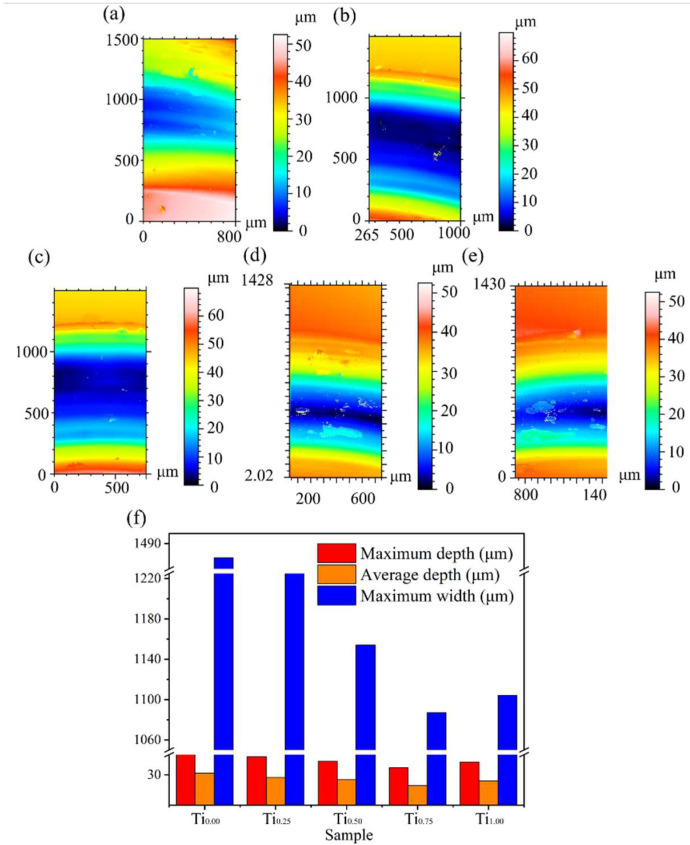
Three-dimensional contours topographies of the worn scars for the CoCrFeMnNiTi_x_ HEA coatings: (**a**) Ti_0_, (**b**) Ti_0.25_, (**c**) Ti_0.5_, (**d**) Ti_0.75_, (**e**) Ti_1_, (**f**) maximum depth, average depth, and maximum width of the wear scar surface area.

**Figure 9:**
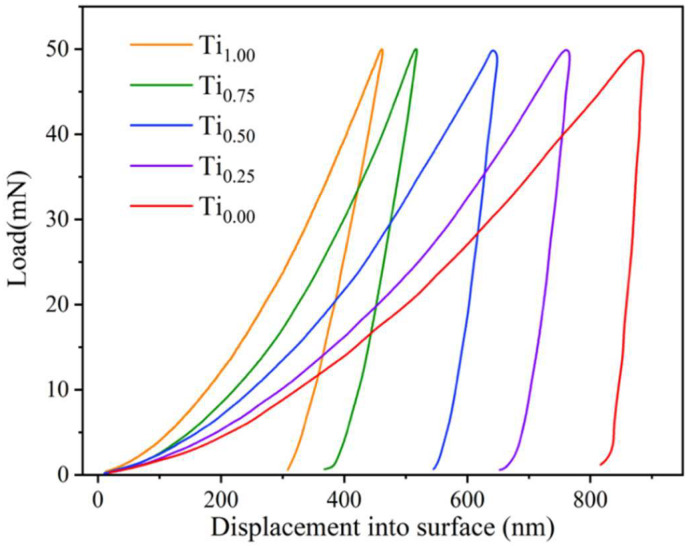
Load–displacement curves of the CoCrFeMnNiTi_x_ HEA coatings.

Figure 8a–e illustrates the three-dimensional contour topography of the wear surface of the CoCrFeMnNiTi_x_ coatings. Combined with Figure 8f, the wear depth and wear mark width decreased first and then increased with the increase in Ti content. The maximum wear width and depth of the Ti_0.75_ coating were 1087 and 39.12 μm, respectively, and the wear resistance was relatively best. Figure 9 demonstrates the load–displacement curve of the nanoindentation test. The displacement decreased first and then increased with an increase in Ti content. As the hardness value of the Ti_0.75_ coating was the largest, the indentation depth reached the lowest value at this time. Table 3 shows the hardness value (*H*) and elastic modulus (*E*) of the indentation characteristic parameters corresponding to different Ti contents. Because *H/E* and *H*^3^*/E*^2^ are related to wear amount, *H/E* is used to evaluate the limit of elastic behavior in surface contact, and *H*^3^*/E*^2^ is the resistance of the material to plastic deformation. According to the experimental results in Table 3, the introduction of Ti can substantially enhance *H/E* and *H*^3^*/E*^2^, and the values of *H/E* and *H*^3^*/E*^2^ of the Ti_0.75_ coating were 1.29 and 3.32 times that of the Ti_0_ coating, demonstrating that the Ti_0.75_ coating has outstanding high plastic deformation ability because Ti intensifies the inherent lattice distortion effect of HEAs and induces grain shrinkage, thus enhancing the coatings’ mechanical properties. Furthermore, the random distribution of the Fe_2_Ti and Ni_3_Ti intermetallic compounds enhanced the coating’s strength and weakened the plastic deformation during friction.

**Figure 10:**
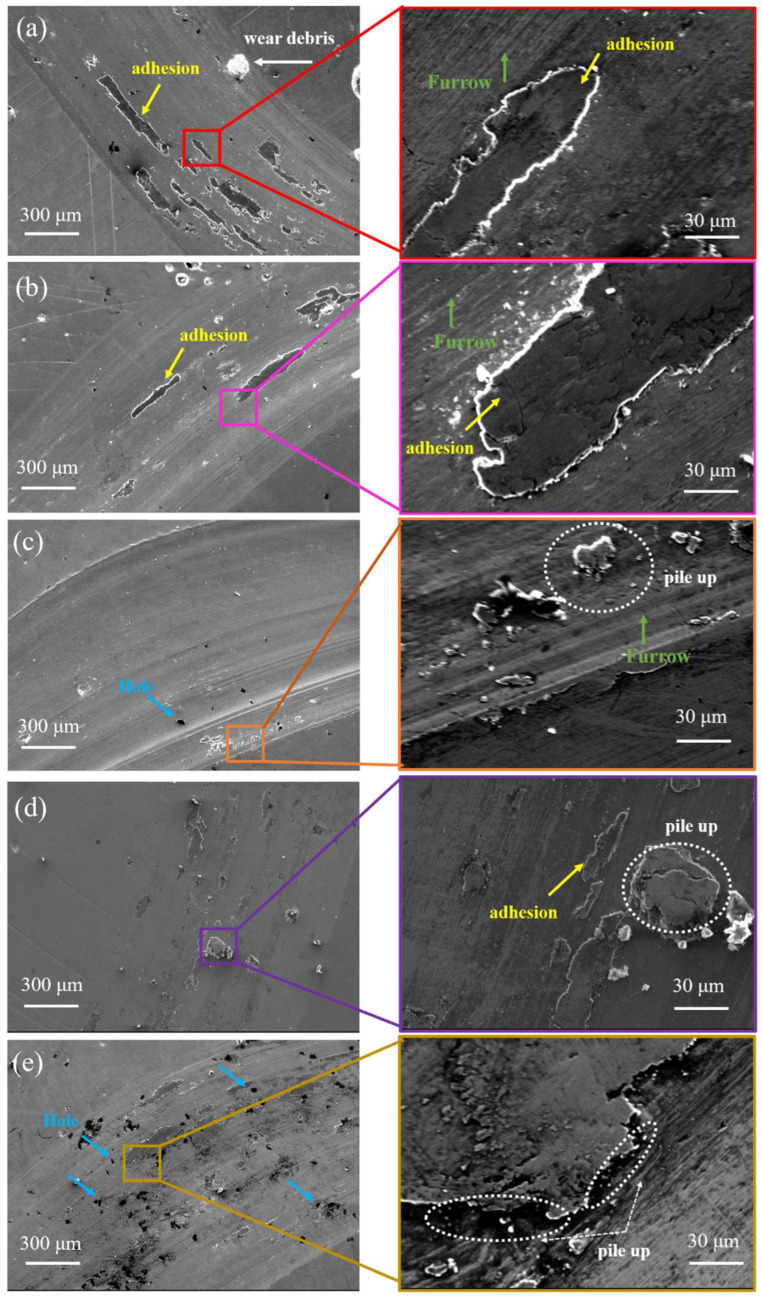
SEM local images of the wear surface of the CoCrFeMnNiTi_x_ high-entropy alloy coatings: (**a**) Ti_0_, (**b**) Ti_0.25_, (**c**) Ti_0.5_, (**d**) Ti_0.75_, and (**e**) Ti_1_.

Figure 10 shows the SEM views of the wear marks of the CoCrFeMnNiTi_x_ HEA coatings. The wear marks were circular and visible, and the surface was distributed with several furrows, adhesion, cracks, chips, and small holes. The abrasive chips and furrows on the worn surface were caused by the continuous plowing of the Si_3_N_4_ ball in the friction process, which is a typical abrasive wear mechanism. The Ti_0_ coating’s wear surface produced a certain plastic deformation under normal load, resulting in the accumulation of materials at the edge of wear marks, and there was obvious plastic deformation and adhesion, indicating the adhesion wear mechanism. The furrow marks on the wear surface became shallow with the increasing Ti content, and the adhesion area (black area) displayed a decreasing trend. As shown in Figure 10b, the adhesion wear of the Ti_0.25_ coating was enhanced. The increase in the coating’s hardness can strengthen the resistance to the dual sample’s plowing effect. As shown in Figure 10c–e, when the molar ratio of Ti increased from 0.5 to 1, the dual sample’s contact cyclic stress in the friction process resulted in the generation of microcracks on the wear surface, which eventually cause material to pile up due to the aggregation of cracks. When x = 0.75, the coating’s strength and hardness were enhanced because of the increase in Ti content-intensified lattice distortion, solid solution strengthening effect, and precipitation strengthening of intermetallic compounds Fe_2_Ti and Ni_3_Ti, and the abrasive wear effect was substantially reduced. A local flake failure zone was formed on the coating’s surface, indicating the existence of a fatigue wear mechanism. Thus, the wear mechanism of the CoCrFeMnNiTi_x_ HEA coating at room temperature is primarily abrasive wear, and adhesion wear and fatigue wear mechanisms exist simultaneously.

## 4. Conclusions

In this study, CoCrFeMnNiTi_x_ HEA coatings were fabricated using an IPG8000 fiber laser on the surface of 40Cr steel. The coatings’ microstructure and tribological properties were investigated. The main conclusions are summarized as follows:

(1) The coatings’ surface was well-formed, and the junction with the base material showed metallurgical bonding. The microstructure of the CoCrFeMnNiTi_x_ HEA coatings showed a typical dendrite structure. With the increase in Ti content, Co, Ni, and Ti were primarily enriched in the interdendrite, Cr was primarily enriched in the dendrite, and the Ti_0_ and Ti_0.25_ coatings maintained a single FCC solid solution phase. With the increase in Ti content, the Ti_0.5_, Ti_0.75_, and Ti_1_ coatings were primarily composed of FCC phases with Fe_2_Ti, Ni_3_Ti, and Co_2_Ti intermetallic compounds.
(2) The microhardness of the coating increased significantly because of the lattice distortion effect, solid solution strengthening effect, and precipitation strengthening effect of the intermetallic compound introduced by Ti with a relatively high atomic radius. The maximum hardness of the Ti_0.75_ coating was 511 HV0.5, which was 1.46 times that of the Ti_0_ coating at 348 HV0.5.
(3) With the increase in Ti content, the friction coefficient and wear loss decreased first and then increased. The lowest values of the Ti_0.75_ coating were 0.296 and 0.001 g. When the x value reached 0.25 (Ti_0.25_), the coating’s adhesion and abrasive wear mechanisms remained unchanged, the coating surface’s adhesion degree decreased visibly, and the wear resistance was effectively enhanced. The wear marks of the CoCrFeMnNiTi_x_ (x = 0.5, 0.75, and 1) high-entropy alloy coatings showed visible stratification and lamellar morphology. With the increase in Ti content, the coating’s wear mechanism changed from adhesive wear and abrasive wear to fatigue wear and abrasive wear.

## Figures and Tables

**Table 1:** Chemical composition of CoCrFeMnNiTi_x_ (at.%).

Sample	Co	Cr	Fe	Mn	Ni	Ti
CoCrFeMnNiTi_0_ (Ti_0_)	17.47 ± 0.03	20.06 ± 0.13	16.81 ± 0.03	28.98 ± 0.11	16.68 ± 0.05	-
CoCrFeMnNiTi_0.25_ (Ti_0.25_)	12.77 ± 0.05	21.02 ± 0.10	16.01 ± 0.19	31.43 ± 0.22	13.99 ± 0.14	4.76 ± 0.02
CoCrFeMnNiTi_0.5_ (Ti_0.5_)	12.19 ± 0.04	20.06 ± 0.17	15.28 ± 0.11	30.03 ± 0.08	13.35 ± 0.19	9.09 ± 0.02
CoCrFeMnNiTi_0.75_ (Ti_0.75_)	11.66 ± 0.06	19.19 ± 0.05	14.61 ± 0.12	28.69 ± 0.11	12.77 ± 0.07	13.05 ± 0.08
CoCrFeMnNiTi_1_ (Ti_1_)	11.17 ± 0.16	18.39 ± 0.05	14.01 ± 0.10	27.50 ± 0.04	12.24 ± 0.11	16.67 ± 0.09

**Table 2:** Chemical composition of dendrite and interdendrite (at.%).

Alloy	Region	Element Content (at.%)							
		Co	Cr	Fe	Mn	Ni	Ti	C	O
Ti_0_	Interdendrite 1	18.62 ± 0.87	19.03 ± 1.34	18.80 ± 2.43	19.25 ± 1.32	19.58 ± 2.17	-	1.32 ± 0.02	3.4 ± 0.03
	Dendrite 1	20.41 ± 0.62	19.88 ± 1.63	20.17 ± 0.97	17.29 ± 1.33	18.25 ± 1.22	-	1.80 ± 0.13	2.18 ± 0.12
Ti_0.25_	Interdendrite 2	19.23 ± 0.53	18.13 ± 0.67	18.76 ± 1.13	17.48 ± 0.98	20.01 ± 1.41	4.99 ± 0.21	1.03 ± 0.03	0.38 ± 0.07
	Dendrite 2	18.06 ± 1.31	20.80 ± 0.42	18.31 ± 0.68	18.10 ± 1.73	19.27 ± 2.62	3.43 ± 0.14	1.3 ± 0.09	0.73 ± 0.02
Ti_0.5_	Interdendrite 3	23.16 ± 0.33	16.37 ± 1.87	16.42 ± 0.85	13.28 ± 1.71	20.56 ± 2.56	9.34 ± 0.37	0.52 ± 0.03	0.35 ± 0.04
	Dendrite 3	17.08 ± 2.44	22.69 ± 2.68	18.54 ± 1.63	18.13 ± 3.21	20.51 ± 1.88	2.29 ± 0.6	0.4 ± 0.17	0.36 ± 0.02
Ti_0.75_	Interdendrite 4	21.31 ± 1.21	15.24 ± 1.52	13.44 ± 1.23	13.06 ± 2.31	22.83 ± 1.67	13.07 ± 0.28	0.39 ± 0.08	0.21 ± 0.03
	Dendrite 4	18.28 ± 0.55	21.68 ± 1.27	19.73 ± 0.91	17.61 ± 2.62	18.94 ± 3.31	2.86 ± 0.12	0.34 ± 0.06	0.56 ± 0.11
Ti_1_	Interdendrite 5	23.31 ± 0.95	10.27 ± 1.31	15.41 ± 0.63	13.16 ± 1.71	20.79 ± 3.11	14.32 ± 0.4	0.58 ± 0.03	0.71 ± 0.06
	Dendrite 5	17.67 ± 0.32	22.19 ± 2.89	19.53 ± 1.53	16.84 ± 0.97	19.91 ± 2.41	2.26 ± 0.1	0.49 ± 0.12	1.01 ± 0.03

**Table 3:** Nanoindentation characteristics of CoCrFeMnNiTi_x_ coatings.

Sample	*H* (Gpa)	*E* (Gpa)	*H/E*	*H*^3^/*E*^2^ (Gpa)
Ti_0_	2.87 ± 0.48	198.51 ± 6.45	0.0144	0.00059
Ti_0.25_	3.62 ± 0.29	233.94 ± 4.8	0.0154	0.00086
Ti_0.5_	4.57 ± 0.51	258.28 ± 2.67	0.0165	0.00143
Ti_0.75_	5.67 ± 0.99	304.35 ± 4.91	0.0186	0.00196
Ti_1_	5.39 ± 0.21	316.31 ± 1.26	0.0170	0.00156

## Data Availability

Data are contained within the article.
